# Modeling search movements of an insect's front leg

**DOI:** 10.14814/phy2.13489

**Published:** 2017-11-17

**Authors:** Tibor I. Tóth, Eva Berg, Silvia Daun

**Affiliations:** ^1^ Heisenberg Research Group of Computational Neuroscience – Modeling Neuronal Network Function University of Cologne Cologne Germany; ^2^ Karolinska Institute University of Stockholm Stockholm Sweden; ^3^ Institute of Neuroscience and Medicine (INM‐3) Research Center Jülich Jülich Germany

**Keywords:** locomotion, motor control, neuromuscular model, stick insect

## Abstract

Beside locomotion, search movements are another important type of motor activity of insects. They are very often performed by the front legs of the animals. They consist of cyclic stereotypical leg movements that can be modified by sensory signals. The details of the local organization of these movements have however not yet been studied. In this paper, we, using an appropriate variant of our existing one‐leg model, present a scheme of how these searching movements might be organized and performed on the level of local neuromuscular control networks. In the simulations with the model, we attempted to mimic the experimental results by Berg et al. (J. Exp. Biol. 216:1064–1074, 2013) in which an obstacle was put in the way of the search movements of the front leg for a very short while, and then the recovery to the usual search movements was observed and analyzed. Our simulation results suggest that the recruitment of the fast levator and depressor muscles play a crucial part in resuming the search movements after removal of the obstacle. The interplay between the levator and depressor, and the extensor and flexor local control networks can, according to the model, bring about a large variety of search movements upon removal of the obstacle. A number of these movements are comparable with those seen in the experiments.

## Introduction

Search movements are, beside locomotion, the other characteristic leg movement type of insects. They are most often carried out by the front legs of the animals. The stick insect can perform them, while it continues walking on its four other legs. The result is a complex three‐dimensional movement of the front legs. Search movements in different insects have been studied by a number of authors: in stick insects (Karg et al. 1991; Bässler, 1993; Dürr, 2001; Bläsing and Cruse, 2004a,b; Schütz and Dürr 2011), in locusts (Pearson, 1972), cockroaches (Delcomyn, 1987), and in fruit flies (Pick and Strauss, 2005). Here, the work by Dürr (2001) should especially be mentioned. He carried out detailed investigations on the three‐dimensional search movements of the stick insect's legs.

However, it is quite difficult to study the details of searching leg movements in three dimensions, in a freely moving animal. In many experiments, the movement of a front leg is therefore restricted. The constraint most often used is that the front leg is only allowed to move in a plane perpendicular to the body's longitudinal axis (e.g., Berg et al., 2013). A protractor–retractor movement becomes thus impossible. However, vertical movements of the femur controlled by the levator‐depressor local neuromuscular network, and movements of the tibia that are governed by the local extensor‐flexor neuromuscular network remain unaffected by this constraint. This constrained system is much easier to study than the unconstrained one. Berg et al. (2013) used this arrangement for their experimental investigation. Adhering to this arrangement, we also used a restricted one‐leg model in our modeling studies (Tóth et al. 2013a).

Our main aim in this study was to use the experimental results, mainly by Berg et al. (2013), and our existing one‐leg model of a stick insect (Tóth et al., 2013a,b) to mimic the search movements seen in the experiments. We formulated a hypothesis on the role the specific local networks of the model might play during search movements. We implemented it as specific properties of the model, and tested it in simulations. Achieving good qualitative agreement between experimental and simulation results, we could gain insight into the workings and coordination of the local neuromuscular control networks, which shape the movements during search.

In what follows, we first introduce the model used in these investigations. We then describe the results, we obtained with the model tailored to the task of simulating search movements. Finally, we shall discuss the merits and shortcomings of our approach and model.

## Methods

### Summary of the experiments

Here, we give a summary of the experiments by Berg et al. (2013). For details, refer to the aforementioned paper.

**Figure 1:**
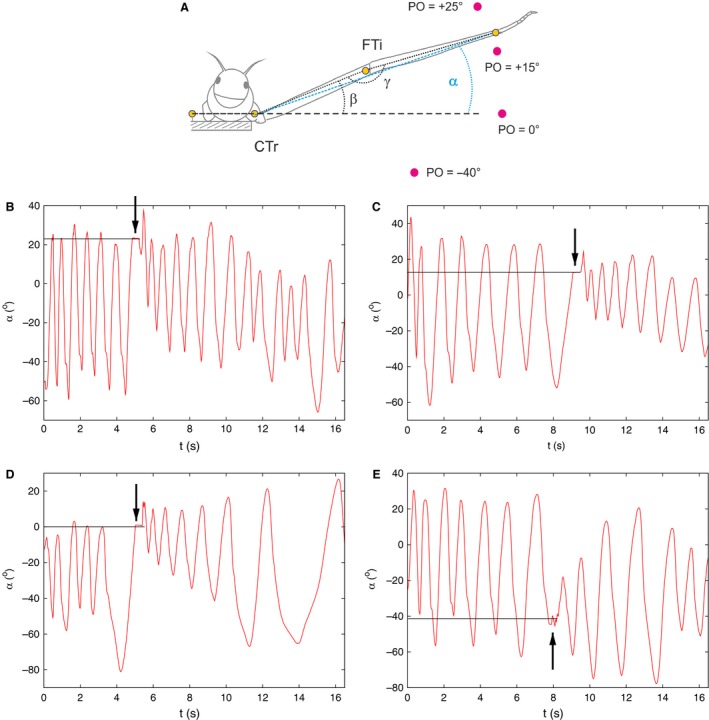
(A) Schematic illustration of the experimental arrangement. (Original Fig. 1C from Berg et al. (2013) with permission.) Note the definitions of the angles *α*,* β* and *γ* in the panel. The large dots labeled with PO = +25^∘^ etc. are the positions of the obstacles applied to the leg movements. The number indicates the angular position of the obstacle with respect to the horizontal axis. CTr, coxa‐trochanter joint; FTi, femur‐tibia joint. (B–E) Typical recovery time courses of the leg movement to the initial search movement after putting an obstacle in the way of the leg tarsus for a very short period of time. The arrows in the panels (B–E) indicate the instant of time of hitting the obstacle by the insect's leg. The horizontal line segments show the angular position of the obstacle (e.g., +25^∘^ in B). Note that the transition depends on the angular position of the obstacle. Modified from Berg et al. (2013), Figure 2, with permission.

Figure 1A illustrates the basic experimental arrangement. The thorax‐coxa joint of the front leg of the stick insect was fixed such that the leg could only move in a plane perpendicular to the longitudinal axis of the animal. However, no constraints were applied either to the coxa‐trochanter joint or to the femur‐tibia joint. Thus, the animal could freely carry out up and down movements and could flex or extend its tibia in the vertical plane just described.

In the experiments, the angles *α*,* β* and *γ*, as indicated in Figure 1A, were measured. Note that they fully characterize the leg movements. Moreover, the angle *α* is not an independent quantity. It can be computed from the two other angles and the lengths of the femur and tibia. Nevertheless, it was often used for describing the leg position in the experiments, as it clearly identifies the angular position of the tarsus (end of the leg). The leg carried out periodic (search) movements before an obstacle at a given position was put in its way (Fig. 1A) for a very short period ( < 100 ms). Upon removal, the recovery to the periodic movements, that is, the transitional movements of the leg were recorded in the experiments. Four examples of this process are displayed in Figure 1B–E. As these panels illustrate, the properties of the transitional movements depended on the position of the obstacle. They were thus indicative of the neuromuscular control mechanisms that brought about those transitional movements. These panels also show that the contact of the tarsus with the obstacle was indeed very short (arrows in Fig. 1B–E). The experimental records supplied for comparison with the simulation results were selected to be of good technical quality but otherwise basically random.

### The one‐leg model of the stick insect

**Figure 2:**
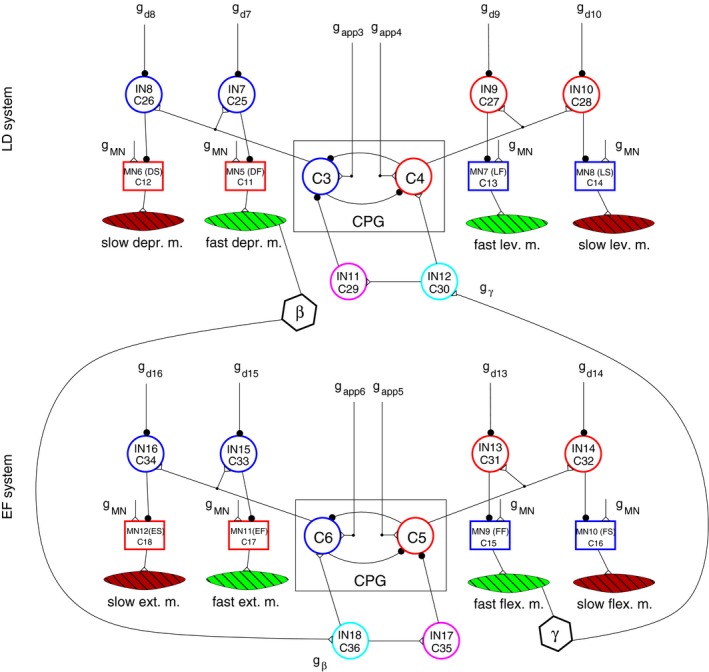
The reduced model. Both local networks, the LD and EF neuromuscular systems, have the same structure as follows. Box with label CPG, central pattern generator consisting of two neurons (C3–C4) and (C5–C6), respectively. The CPGs are (centrally) driven by the excitatory synaptic connections *g*_app3_–*g*_app4_ and *g*_app5_–*g*_app6_, respectively. Four motoneurons (MNs): MN(LF), MN(LS), MN(DF), MN(DS) and MN(EF), MN(ES), MN(FF), MN(FS), respectively, drive the corresponding fast and slow muscles: fast depr. m., slow depr. m. etc. as indicated. The MNs are uniformly centrally driven by the excitatory connections *g*_MN_. Four inhibitory premotor interneurons in each of the networks: IN7–IN10 in the LD system, and IN13–IN16 in the EF system. They are individually inhibited by the (central) synaptic connections *g*_d7_–*g*_d10_ and *g*_d13_–*g*_d16_. Finally, the activity of the interneurons IN11–IN12 directly affects that of the CPG neurons (C3, C4) in the LD system. In the EF system, the interneurons IN17 and IN18 have an analogous function. At synapses, empty triangles mean excitatory, whereas filled circles inhibitory synaptic connections. Greek letters *β* and *γ* in hexagons: sensory signals between the local networks encoding position; *g*_*β*_ and *g*_*γ*_: actual values of synaptic conductances on IN18 and IN12, respectively, generated by the corresponding sensory signals. Adapted from Tóth et al. (2013a) with permission.

In this work, we used a reduced version of our existing model of a single leg of the stick insect. The full model was originally conceived to elucidate the role of fast and slow muscles during locomotion and maintaining the posture (Tóth et al. 2013a). It comprises the three main antagonistic muscle pairs of the leg: the protractor–retractor (PR), levator‐depressor (LD) and extensor‐flexor (EF) muscle pairs, which are the most important for movements of the leg. They are controlled by a local network each (PR, LD and EF system). These networks are coupled by sensory signals from the LD system (*β* signals) and from the EF system (*γ* signals). In the model, they are somewhat abstract representing both position and load signals. As the leg's horizontal position was fixed in the experiments by Berg et al. (2013), and the PR system thus rendered ineffective, we could reduce the model by omitting the PR system from it. The resulting reduced model is displayed in Figure 2. This model contains both fast and slow muscle fibers and the corresponding motoneurons (MNs) that drive them. The activity of the MNs, in turn, is controlled by a uniform central drive (*g*_MN_) and the (rhythmic) signals of the central pattern generators (CPGs) of each local network. The latter signals pass through and can be modified by the inhibitory premotor interneurons (INs) such as IN7, IN8 etc. The activity of these INs is controlled by (descending) inhibitory synaptic inputs (*g*_d7_, *g*_d8_ etc.). The local networks (LD and EF system) are coupled via the sensory signals *β* and *γ* encoding position. Their synaptic pathways converge on INs (IN12, IN18) that directly affect the actual function of the corresponding CPG via the conductances *g*_*β*_ and *g*_*γ*_ (Fig. 2). By appropriate tuning of the coupling mechanism, the model can attain a state of sustained rhythmic activity, which would correspond either to normal locomotion or to search movements. In the former case, sensory signals representing load and ground contact and generated by the tibial campaniform sensilla are also present (Zill et al. 2004, 2011). The central inputs to the CPG (*g*_app3_ etc.) and to the premotor inhibitory INs (*g*_d7_ etc.) are kept constant. We implemented the coupling in this model in a simple way: the conductances *g*_*β*_ and *g*_*γ*_ had either a “high” or a “low” value. The transition from “high” to “low” or in the other direction took place at a threshold value of *β* and *γ*, respectively. Thus the function of the model crucially depends on the choice of these threshold values. (We denote them *β*_thr_ and *γ*_thr_, respectively.) They therefore play a central role throughout this study. We also included muscle recruitment into the model for muscles where it made sense (especially for the fast levator and depressor muscle pair) (Tóth et al., 2013a) based on experimental results by Goldammer et al. (2012). Further details concerning this model can be found in Tóth et al. (2013a,b).

## Results

In the experiments in which the front leg movement was artificially restrained in the vertical plane perpendicular to the longitudinal axis of the body, we found that the angle *α* varied in the range [−80°,40°], *β* in [−30°,50°], and *γ* in [70°,170°]. These angles were defined as shown in Figure 1A. In the simulations, we tried to produce similar ranges for the individual angles. Thus we typically had the range [−50°,20°] for *α*, [−5°,30°] for *β*, and [85°,170°] for *γ*. These ranges are somewhat narrower than those in the experiments. We could have had a better approximation of the angular ranges seen in the experiments, if we had changed some mechanical properties of the muscle fibers (e.g., minimal length, range of spring constants, contraction kinetics etc.). But this would have come with the loss of the ability of the model to produce “normal” stepping behavior with these new parameter values. By this, we would, in fact, have had two models: one for stepping and another for search movements. To avoid this, we simply used the angular ranges, which the “stepping” model yielded.

In any case, our main aim was to achieve good agreement of the *qualitative* properties of the experimental and simulation results. At first sight, this may seem a rather weak requirement but closer inspection shows that it has been the proper choice in our case. Firstly, the same mechanisms can, and probably would, produce different quantitative results, if the values of (some of) the system parameters differ. These values are certainly different in the animal and the model, and may not even correspond to each other. At the same time, it does not appear viable to construct a model in which the correspondence between the parameters of the animal and the model is strong, let alone that the parameter values are approximately equal. Secondly, even more importantly, one often tacitly accepts models to be quantitative ones, whereas, in fact, they are qualitative ones because the simulation results they produce differ in a number of *quantitative* characteristics. Yet, they are accepted, and rightly so, since the results produced by them, carry the hallmark of the behavior of the original system (e.g., activity of a neuronal network). For these reasons, we used *qualitative* criteria, only, to judge whether our simulation results were acceptable. We think that we could achieve sufficiently good approximations of the experimental results by means of our model, using the original value set of its parameters.

Moreover, we identified four main physiological factors that have a bearing on the search movements and their recovery. These are (1) the workings of the fast or slow muscles during these movements; (2) the activity patterns and recruitment of the MNs driving these muscles; (3) the flow of sensory signals between the LD and EF local control networks; and (4) the properties of the couplings between them, in particular, the threshold values *β*_thr_ and *γ*_thr_ of the angles *β* and *γ* at which the coupling conductances *g*_*β*_ and *g*_*γ*_ change their values (cf. Methods).

Making use of the main physiological factors just listed, we formulated the following hypothesis of how the recovery of the search movements might take place in the stick insect and what mechanisms might participate in this process. We implemented the details in the model and tested them by using simulation results produced by the reduced model. Thus our hypothesis on the recovery process is as follows:

1. Before hitting the obstacle, both the slow and fast muscle fibers are active, that is, both carry out rhythmic contractions but the activity of the fast muscle fibers determine the search movement.
2. Upon hitting the obstacle, the activity of all slow and fast MNs ceases. Note that because of the inherent rigidity of the muscles (Hooper et al. 2007), the position of the leg remains constant for approximately 100 ms.
3. After removal of the obstacle, all slow muscle fibers become activated via the activation of their MNs within 200 ms. The fast extensor and flexor MNs, hence muscles are immediately activated. Since there is just a single slow and a single fast extensor MN (Bässler and Storrer, 1980; Goldammer et al. 2012), a gradual increase of the muscle activity by recruitment of more and more MNs is not possible.
4. Most importantly, the fast levator and depressor muscle fibers and their MNs are *gradually* recruited over a longer period of time, which can reach more than 1 sec of duration. Gradual recruitment of the MNs and muscle fibers is, in this case, possible, because there are several fast levator and depressor MNs (Goldammer et al. 2012). The time course of the recruitment of these muscles depends on the angular position of the obstacles.

The experimental records in Figure 1B–E seem to support this hypothesis but at least they do not contradict it. They show a gradual shift of the oscillatory minima after removal of the obstacle depending on the obstacle's position, and a gradual increase of oscillatory amplitude in the vertical plane.

We implemented the properties and mechanisms listed in the hypothesis. Some of them had been part of the original model, especially the properties of the slow and fast muscles and their residual rigidity (Tóth et al. 2013a).

**Figure 3:**
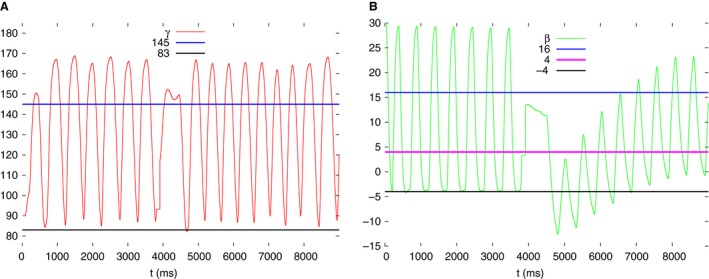
Values of *γ*_thr_ and *β*_thr_ used in the simulations are related to the angles themselves. (A) two values of *γ*_thr_: 83^∘^, 145^∘^ and the time course of the angle *γ*, as indicated in the panel. (B) three values of *β*_thr_: −4^∘^, 4^∘^, 16^∘^, and the time course of the angle *β*, as indicated in the panel. Note that in A the line *γ*_thr_ = 83^∘^ hardly intersects the time course of *γ*.

Since a theoretical approach is, because of the complexity of the model, not practicable, we carried out simulations to determine the range of threshold values of *γ* and *β* at which the model displays autonomous rhythmic (periodic) activity. We found two disjoint intervals: *γ*_thr_≤86°, and 144°≤*γ*_thr_≤149°. The two ranges implied two qualitatively different driving mechanisms for the rhythmic (periodic) leg movements (see below). In the former case, since the oscillations of the *γ* angle hardly cross into the region *γ*_thr_≤86° Fig. 3A), no switch to the low value of *g*_*γ*_ can take place. Hence, *g*_*γ*_ permanently remains at its high value, and the LD system receives steady excitation via IN12 (Fig. 2). In the latter case (144°≤*γ*_thr_≤149°), periodic switching between low and high values of *g*_*γ*_ takes place during the leg movements (Fig. 3A). Thus, the excitation the LD system receives via IN12 is also periodic. We found that we could obtain best simulation results if we used different recruitment kinetics for high threshold values of *β* (*β*_thr_ > 10°) and low ones (*β*_thr_≤6°). This proved especially important when the second range of *γ*_thr_ (144°≤*γ*_thr_≤149°) was used in the simulations. Here, the range of high *β*_thr_ values at which periodic leg movement was produced was narrow: [14.5°≤*β*_thr_≤16°]. No restriction applied to the low *β*_thr_ values in the interval [−4°,6°]. The *β* threshold values were not subjected to constraints, if *γ*_thr_ was selected from the first range (*γ*_thr_≤86°) of threshold values. One can easily guess that this is because of the permanent excitation of the LD system by the EF system via IN12. In Figure 3A, two values of *γ*_thr_ are displayed, along with the time evolution of *γ* during periodic leg movements, that we used throughout the simulations (*γ*_thr_ = 83°, 145°). Similarly, Figure 3B shows *β*(*t*) with the three values of *β*_thr_ = −4°, 4°, 16° used in the simulations. The actual threshold values were chosen such that the simulation yielded the best (qualitative) approximation of the experimental results at a given angular position of the obstacle.

**Figure 4:**
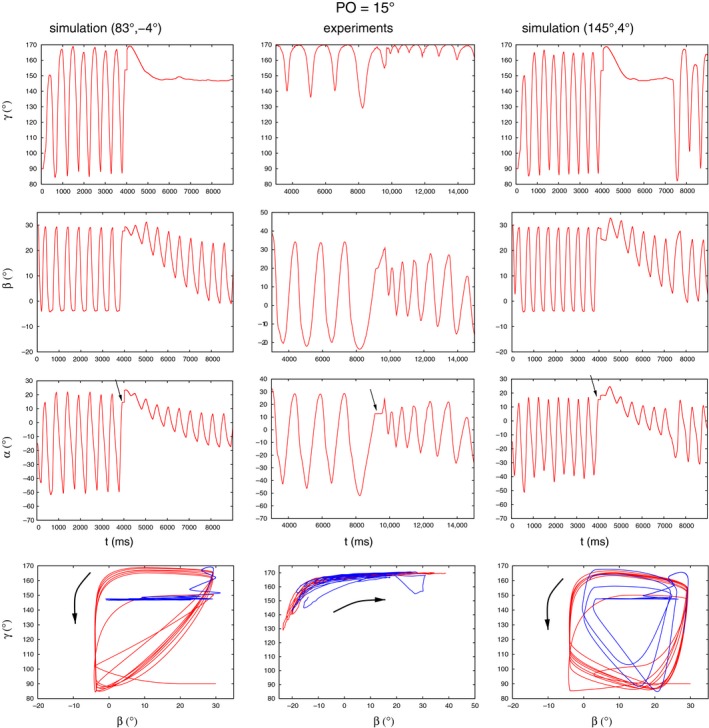
Comparing experimental results (middle column of panels) to simulation ones with two different sets of threshold values (left and right columns of panels) at an obstacle position of 15^∘^. The time courses of the angles *α*,* β* and *γ* are illustrated in the rows of panels as indicated. Bottom row of panels: *β*−*γ* state diagrams. The red trajectories apply before placing the obstacle, the blue ones after its removal. We use the notation “simulation (*γ*_thr_, *β*_thr_)” to show which threshold values were used in the simulations. Note that the time interval is somewhat longer, and the angular ranges somewhat larger in the experiments than in the simulations. That is, the simulated angle variables oscillate faster and with smaller amplitudes than the experimental ones. The arrows in the *α* panels point to the angle position at the time instant of the leg (tarsus) hitting the obstacle. The arrows in the *β*−*γ* state diagrams show the direction of time the trajectories follow. Note that they are the same in the experiment and the simulations.

In the following, we shall present the corresponding simulation results. Figure 4 displays the first such example. In this figure, the time course of all three angles, *γ*,* β*, and *α* both in the experiment (middle column) and in the simulations (left and right columns) are shown. In the bottom row of panels, the state diagrams *β*−*γ* obtained in the experiments and the simulations are exhibited. The time courses of the angles *α* and *β* of both simulations show close *qualitative* similarity to that of the experimental traces. Both angles show small‐amplitude oscillation upon removal of the obstacle, where the amplitude of the oscillations increases with time. This is a property of both the experimental and the simulated time courses. Taking these properties alone, both simulations appear to yield satisfactory results, even though the threshold values used in them are completely different. We encounter, to some extent, a similar situation when inspecting the behavior of the angle *γ*. Here, *γ* changes very little after removal of the obstacle in the experiment, at least in the interval shown, (Fig. 4, middle column), and permanently remains (almost) constant (Fig. 4, left column) or, at least, for a longer period of time (Fig. 4, right column) in the simulations. Since the value of *γ* at which this happens is about 150°, the leg is in a stretched position. Only its vertical position changes all the time (because of oscillating *β*). Thus the qualitative agreement between experiment and simulation, here too, appears to be satisfactory. Now, considering the state diagrams in the bottom row, it should be noted that in all three trajectories, the direction of time is the same (as indicated by arrows in the three panels). A further common property of theirs is the presence of the (almost) horizontal blue lines, expressing the (almost) constant values of *γ* after removal of the obstacle. At the same time, substantial differences between them can also be discovered. A comparison between the simulation and experimental results therefore remains inconclusive. One can, however, immediately recognize that the two sets of threshold values in the simulations lead to qualitatively different state diagrams. In this case, neither the time courses of the angles nor the state diagrams show which set of parameter values yield better results. We obtained similar results at an obstacle position of 0° with sets of threshold values (*γ*_thr_ = 83°, *β*_thr_ = 4°), and (*γ*_thr_ = 145°, *β*_thr_ = 4°) (not shown).

**Figure 5:**
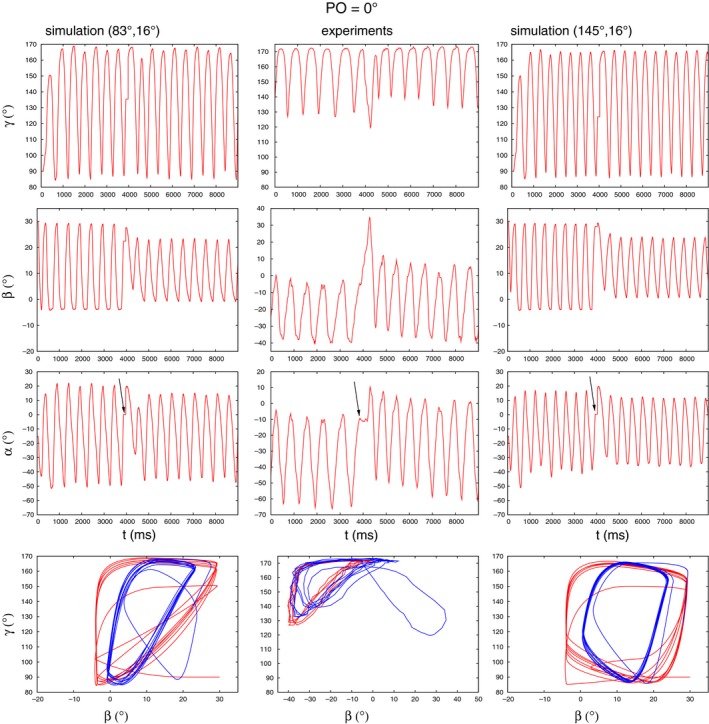
Comparing experimental results (middle column of panels) to simulation ones with two different sets of threshold values (left and right columns of panels) at an obstacle position of 0^∘^. Note that the transition signals do not show large upward or downward trends, neither in the experiment nor in the simulations. Note also the close similarity between the experimental state diagram and the simulated one in the left column. The arrows in the *α* panels point to the angle position at the time instant of the leg (tarsus) hitting the obstacle. The direction of time the trajectories follow in the *β*−*γ* state diagrams is the same as in Figure 4. All notations and the color codes are the same as in Figure 4.

The experimental results varied, even for the same obstacle position, a great deal across the experiments (Berg et al., 2013). Thus, for example, at the obstacle position of 0°, we found several, *qualitatively* different time courses of the angles *α*,* β*, and *γ* in the transition process. We carried out simulations in order to mimic the experimental results that differed from those just mentioned above but were obtained at the same obstacle position (0°). The results are displayed in Figure 5. Here, again judging by the time courses of the angular variables, one cannot decide which simulation results should be preferred to the other. With both sets of threshold values, (83°,16°) and (145°,16°), respectively, we obtained basically the same time courses of the angles *α*,* β* and *γ*. However, the comparison of state diagrams has a different outcome. Here, when setting *γ*_thr_ = 83°, the simulated state diagram exhibits a much closer similarity to the experimental one than the other simulated one. In fact, the experimental state diagram is of a triangle‐like form both before placing the obstacle (red trajectory) and after removing it (blue trajectory). Specifically, both trajectories have an almost horizontal segment that is bent into an almost vertical segment, the points of bending being approximately at the most negative value of *β*. The two endpoints of these segments are then connected by an almost linear curve. Qualitatively, the same properties can be discerned in the simulated state diagram on the left‐hand side, although the absolute ranges of the angles somewhat differ from those found in the experiments. Note that both the experimental and this simulated state diagram preserve their triangular shape after collision with the obstacle. By contrast, the other simulated state diagram (right‐hand side panel) has quite different characteristics: it is roughly of a shape of a quadrilateral, also both before and after the leg touches the obstacle. In this case, the “goodness” of the simulation result can be judged by using the state diagrams rather than the time courses of the angle variables alone.

**Figure 6:**
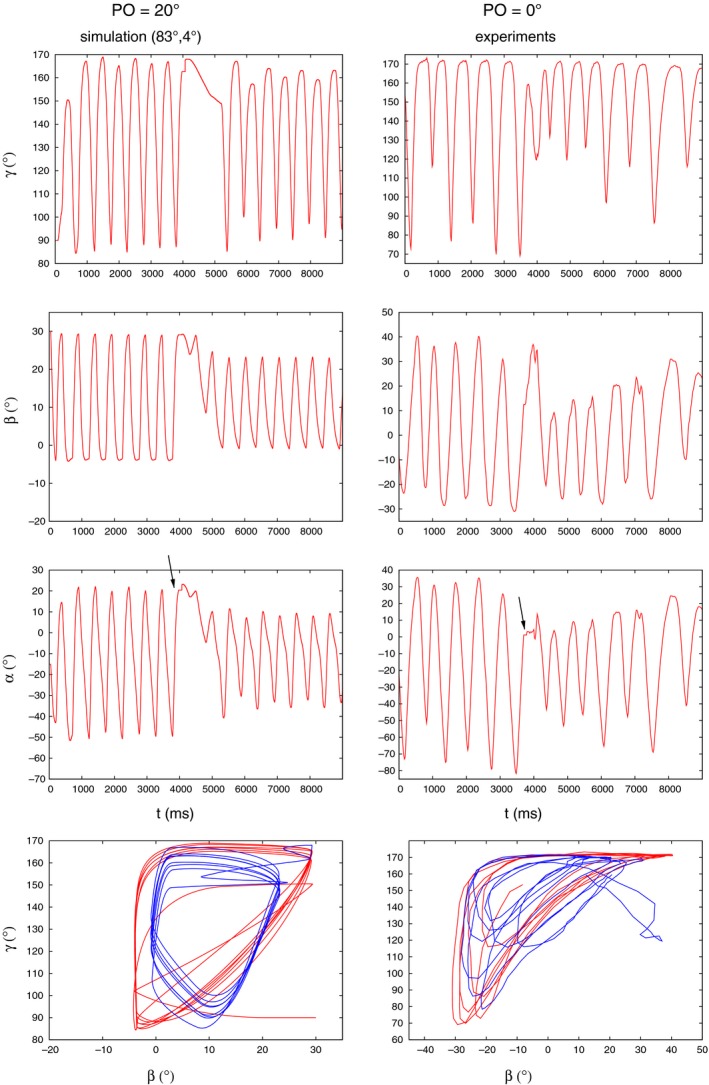
Comparing experimental results (left column of panels) to simulation ones (right column of panels) at different obstacle positions: 20^∘^ and 0^∘^, respectively. Note the oscillation of *γ* with alternating amplitudes, especially after removal of the obstacle. This property is reflected in the oscillation of *α* to a lesser extent. Note also the clear change of the characteristics of the simulated state diagram after removal of the obstacle (from triangle‐like into a quadrilateral‐like shape). This change is not as clear in the experimental state diagram but the initial triangle‐like shape is clearly identifiable. The arrows in the *α* panels point to the angle position at the time instant of the leg (tarsus) hitting the obstacle. The direction of time the trajectories follow in the *β*−*γ* state diagrams is the same as in Figure 4. All notations and the color codes are the same as in Figure 4.

A further example of mimicking experimental results is illustrated in Figure 6. In this case, the simulations carried out with the same obstacle position as in the experiment (0°) did not yield sufficiently good qualitative similarity between experimental and simulation results. We thus looked at simulation results obtained with other obstacle positions. These positions had to remain as close as possible to the experimental one (0°). Under this constraint, we found that the best results were achieved when the obstacle position was 20°. This obstacle position was thus the nearest to 0° among the good simulation results. The peculiarity of the experimental records is that the angles, especially *γ*, exhibit oscillations of alternating amplitudes after removal of the obstacle. This was successfully simulated by the model for *γ* but the aforementioned property is missing from the oscillation of *β* in the simulation. Hence, it is weaker in the simulated oscillation of *α* than in the experimental one. Nevertheless, there is still a sufficient qualitative agreement between the experimental and simulated time courses of the three angle variables. Comparing the state diagrams in the bottom row of Figure 6, we see that they show good agreement before the placement of the obstacle. After removal of the obstacle, the characteristics of the simulated state diagram clearly changes: from the so‐called triangle‐like shape to a quadrilateral‐like one. This kind of change can, however, not be discerned with certainty in the experimental state diagram. We shall return to this point later.

**Figure 7:**
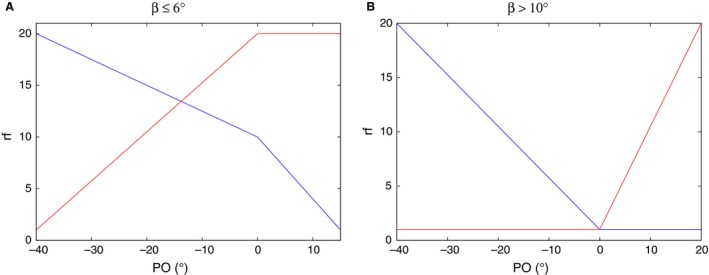
Relationship between obstacle position and recruitment kinetics. Two types of linear relationships between obstacle position (PO) and the so‐called recruitment factor (rf) were used in the simulations. rf expresses the change of the recruitment rate depending on the obstacle position (PO); rf > 1: slow‐down, and rf = 1: “normal” recruitment rate. (A) linear relationship used with low values of *β*_thr_(≤6^∘^); (B) linear relationship used with high values of *β*_thr_( > 10^∘^); red lines: rf of the fast depressor muscle; blue lines: rf of the fast levator muscle.

In the experiments, data could not be collected from every animal at every position of the obstacle. Moreover, there are considerable gaps between the specific obstacle positions where no measurements were done. In a series of simulations, we tried to fill in these gaps. We thus changed the position of the obstacle in the model and computed the time courses of the three angles with six different combinations of threshold values selected earlier: *γ*_thr_ = 83°, 145°;  *β*_thr_ = −4°, 4°, 16°. The main problem to be solved was to connect the obstacle positions to some model properties. At the beginning of this section (Results), we made the assumption as part of our hypothesis, that the recruitment of the fast levator and depressor muscles was involved in the transitional process after removal of the obstacle. In the simulations reported here, we included a quantitative dependence of the rate of recruitment of these muscles on the position of the obstacle. To keep things simple, we assumed linear relationship between the obstacle position and the factor by which the recruitment process of the muscles was slowed down. Thus, the actual value of the recruitment factor of both the fast levator and the fast depressor muscles could be calculated by linear interpolation; formally:

$$\text{rf}=\frac{{\text{rf}}_{1}-{\text{rf}}_{0}}{{\text{PO}}_{1}-{\text{PO}}_{0}}\left(\text{PO}-{\text{PO}}_{0}\right)+{\text{rf}}_{0}$$

where rf is the actual value of the recruitment factor at obstacle position PO. It is the factor of slow‐down of the muscle recruitment during the transition period. By definition, the “normal” recruitment rate occurs at rf = 1. Then at rf > 1, this rate is diminished by the factor rf. For example, rf = 2 means that the recruitment happens twice as slow as at the “normal” recruitment rate (see also Tóth et al., 2013a,b). rf_0_ and rf_1_ are known values of rf at (the known) obstacle positions PO_0_ and PO_1_, respectively. rf_0_ and rf_1_ can be determined (estimated) in the experiments by measuring the duration of the nonzero trend in the transition process (Berg et al., 2013). However, we found qualitatively different behavior of the animal's leg at the same obstacle position for some positions. For example, for PO = 0°, the time courses of *α* in Figure 2C and Figure 4 in Berg et al. (2013) are quite different. Running altogether a few hundreds of simulations with all six combinations of the threshold values *β*_thr_ and *γ*_thr_, and using various linear interpolation functions, we found that the above differing behavior, as well as the cases at other PO values could satisfactorily be modeled by using only two different sets of linear interpolation functions. They are displayed in Figure 7. Thus the leg's behavior as shown in Figure 2C in Berg et al. (2013) could qualitatively be replicated by the linear interpolation functions for rf in Figure 7A and using the low values of *β*_thr_ = −4°,4°. The leg movement in Figure 4 in Berg et al. (2013) could be best described when we used the high value of *β*_thr_ = 16° and the linear interpolation functions displayed in Figure 7B. Using these relationships, we could compute the (simulated) time courses of all three angles at any obstacle position (PO). This enabled us to carry out systematic simulations over the whole range of obstacle positions at various value combinations of *β*_thr_ and *γ*_thr_. As before, we used *γ*_thr_: 145°, 83°, and *β*_thr_:−4°, 4°, 16°. This resulted in a large number, more than 300, simulation runs.

**Figure 8:**
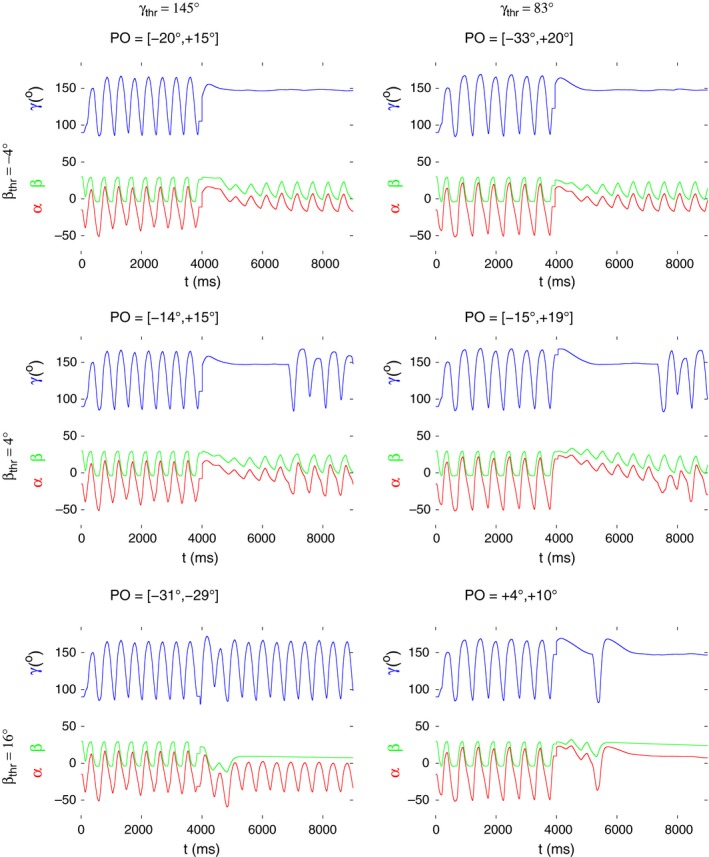
Partial or complete stop of the oscillation of the *β* and *γ* angles upon removal of the obstacle at various combinations of the threshold values *β*_thr_ and *γ*_thr_. These values are displayed above and left of the figure panels, as indicated. Above each panel, the range or value of the obstacle position (PO) is shown where the behavior illustrated in the panel arises. The time courses in each of the panels are as follows; blue: *γ*(*t*), green: *β*(*t*), and red: *α*(*t*). Note that in most of the PO ranges shown, the oscillation of *γ* ceases completely, or at least for a longer period of time. The oscillation of *β* ceases in the interval of POs: [−31^∘^,−29^∘^] (bottom left panel). Finally, at the two following discrete positions: PO = 4^∘^ and PO = 10^∘^, the oscillation of both angles stops.

**Figure 9:**
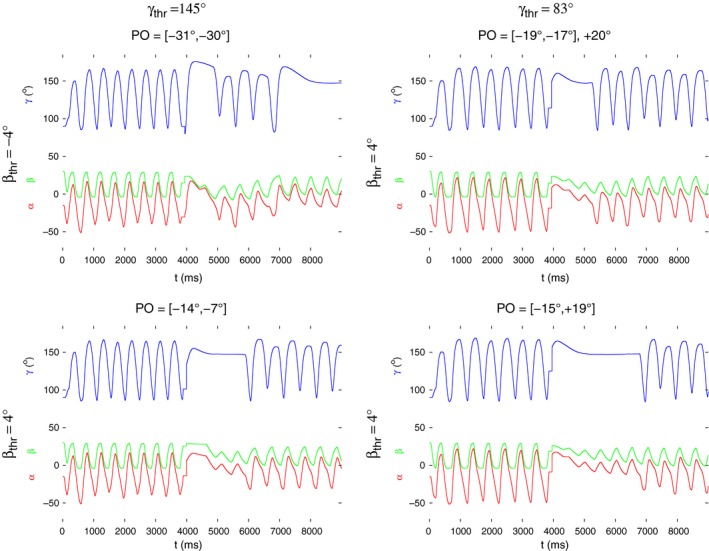
*γ* oscillation with alternating amplitude upon removal of the obstacle at different obstacle positions (PO values). Their sub‐ranges and individual values where this occurs are displayed above each panel. The threshold values at which this type of oscillation can be produced are indicated above and left of each panel. Note that the quiescent period of *γ* before the re‐start of the oscillation varies considerably, depending on the threshold values used. Also the alternating amplitude is reflected differently in the time course of *α*: weak to nonexistent in the panels on the left‐hand side; stronger in those on the right‐hand side.

In the two following figures (Figs. 8 and 9), we illustrate the occurrence of two interesting properties common to several values or sub‐ranges of the obstacle positions. They of course depend on the actual threshold values used in the simulations. In Figure 8, all (PO) intervals are shown in which the oscillation of *γ* (blue traces) or *β* (green traces) ceases completely or at least for a longer period of time at a given pair of *β*_thr_ and *γ*_thr_. As it can be seen, these intervals for *γ* comprise almost the whole range of PO values, if *β*_thr_ = −4° irrespective of the value of *γ*_thr_. They become somewhat shorter when *β*_thr_ = 4°. By contrast, the *γ* oscillation continues but that of *β* stops in a short interval of PO values: [−31°,−29°] if *β*_thr_ = 16° and *γ*_thr_ = 145°. The physical interpretation of these phenomena is quite obvious. If angle *γ* stays nearly at the same position (Fig. 8), only up‐down movements of the leg take place. Interestingly, in all cases in which the *γ* oscillation ceases *γ* is kept at a high value. This corresponds to a stretched leg. One could indeed observe in some experiments up‐down movements of a stretched leg in the stick insect (Berg et al., unpubl. observ.). The permanently constant value of *β* would mean that the tibia carries out periodic extension‐flexion movements at a fixed vertical position, constant *β* angle, of the femur. This was observed in experiments, in those special cases, when the femoral chordotonal organ (fCO) was cut (Karg et al. 1991) but not in intact animals. It may be possible that the sensory signal from the fCO can reversibly be blocked by some appropriate internal neuronal mechanism. In such a case, one would observe this type of leg movement. Finally, we found two discrete values of PO (4° and 10°) at which *both* the *β* and the *γ* oscillation simultaneously ceased (Fig. 8 bottom right panel). This also implies *α* ≈ const., that is, a standstill of the leg (in a nearly horizontal, stretched position). This behavior of a stick insect leg has not been observed. Note also that this happened in the simulation only when *β*_thr_ = 16° and *γ*_thr_ = 83°.

Figure 9 shows a different property of the search movements and of its distribution over the range of the obstacle positions (PO values). In this figure, all *γ* traces have the common property that, after removal of the obstacle and a quiescent period, they exhibit an oscillation with alternating amplitude, that is, a large amplitude is followed by a smaller one, which, in turn, is followed by a larger one and so on. The length of the quiescent period before the restart of the oscillation strongly depends on the threshold values used but, to some extent also on the obstacle positions (cf. the right‐hand side panels of Fig. 9). The ranges of PO values where the *γ* oscillation has these properties are rather small, except for the case in the bottom, right‐hand panel PO: [−15°,19°]. On the other hand, the quiescent period is the longest in this range. As far as the threshold values are concerned, *β*_thr_ = 16° is conspicuously absent. That is, no such *γ* oscillation could be produced in the simulations when *β*_thr_ = 16°. This also means that the interpolation functions in Figure 7A were used throughout these simulations.

Also, we had to use *β*_thr_ = 4° with *γ*_thr_ = 83° in order to achieve the most animal‐like dynamics. It is noteworthy that the angle *β* did not show oscillatory activity with alternating amplitude. However, this type of oscillation is reflected in the time course of the angle *α* in the cases in which *γ*_thr_ = 83° and *β*_thr_ = 4° (Fig. 9, right‐hand side panels) but is absent if *γ*_thr_ = 145°.

**Figure 10:**
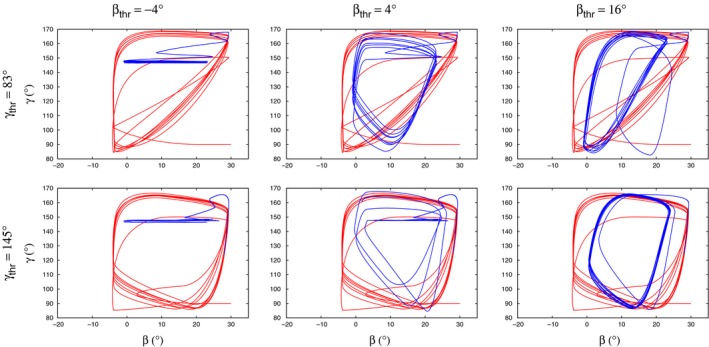
Some typical state diagrams as obtained in the simulations; red: trajectory before placement, blue: after removal of the obstacle. Each pair of threshold values is represented by one example, as indicated in the figure. The value of *γ*_thr_ determines the qualitative shape of the state diagram before placement of the obstacle. The choice of *β*_thr_ can modify that shape in some cases (*β*_thr_ = −4^∘^, left panels; *β*_thr_ = 4^∘^ upper middle panel) after removal of the obstacle. The direction of time for all trajectories is the same as in Figure 4.

We now return to the *β*−*γ* state diagrams. Figure 10 illustrates some typical state diagrams, which we obtained in the simulations. Each pair of threshold values is represented. Inspecting these diagrams, one can gain important information on the role of the threshold values in shaping the state diagrams, hence the activity of the local control networks underlying them. First of all, it can clearly be seen that the shape of the state diagrams before placing the obstacle (red lines) is completely determined by the value of *γ*_thr_ irrespective of that of *β*_thr_. In particular, if *γ*_thr_ = 83°, we obtain the triangular form of the state diagram regardless of the value of *β*_thr_. At *γ*_thr_ = 145°, the shape of the diagram becomes a quadrilateral. Looking at Figure 3A, the reason for the difference between the two cases becomes apparent. *γ*_thr_ = 83° is a threshold value at which the conductance *g*_*γ*_ has a constant high value, hence there is a permanent high‐conductance excitatory input from the EF to the LD system. When *γ*_thr_ = 145°, then *g*_*γ*_ is periodically switched between its high and low value (cf. Fig. 3A). As to the functional role of *β*_thr_, it can substantially change the shape of the state diagram *after removal* of the obstacle (blue lines). Specifically, at *β*_thr_ = −4°, the *γ* oscillation ceases, and the leg continues moving in a vertical plane, while it remains stretched. This happens independently of the actual value of *γ*_thr_. When *β*_thr_ = 4°, a qualitative change of the state diagram occurs at *γ*_thr_ = 83° from the triangular quadrilateral shape, whereas at *γ*_thr_ = 145°, a quiescent period of *γ* appears before the oscillation re‐starts (see also Fig. 9, left bottom panel). Finally, at *β*_thr_ = 16°, no qualitative change of the state diagram occurs, whatever value *γ*_thr_ has. In the aforementioned systematic simulations (Figs. 8 and 9) that used all six selected pairs of threshold values, and the obstacle positions that most often appeared in the experiments, the qualitative properties of the state diagrams could be classified the same way as those of the state diagrams in Figure 10. In particular, the dependence on the threshold values was the same.

**Figure 11:**
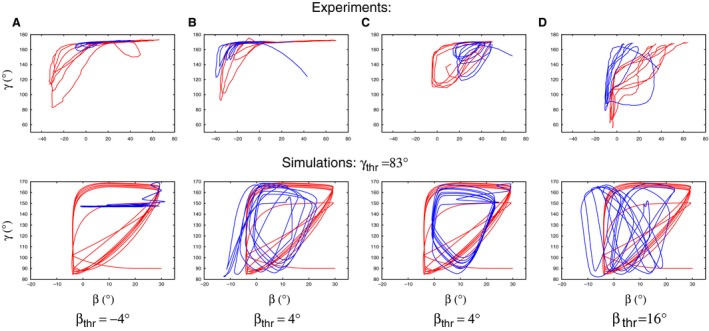
Comparison of experimental (upper row) and simulated (bottom row) state diagrams. The positions of the obstacle are given as follows. (A) 15^∘^, (B) −40^∘^, (C) 25^∘^, and (D) −40^∘^. Suitable simulated state diagrams were selected to have the best qualitative similarity to, and the same or nearly the same PO value as the corresponding experimental diagram. The value of *β*_thr_ at which a simulated state diagram was obtained is indicated at the bottom of each pair of panels; in all cases *γ*_thr_ = 83^∘^. In the experimental and simulated state diagrams, red curves: before placement, blue curves after removal of the obstacle. In the pairs of panels A and C, the experimental and simulated state diagrams show a good qualitative agreement. In A, *γ* oscillation ceases upon removal of the obstacle, in C, the shape of the loop appears to have changed after removal of the obstacle. But also in the panel pairs B and D, a weak resemblance of the experimental state diagrams can still be discerned in the corresponding simulated ones after removal of the obstacle. The direction of time for all trajectories is the same as in Figure 4.

To carry on along these lines, we compared experimental state diagrams with suitable simulated ones having the same, or nearly the same obstacle position as the experimental one. Here, “suitable” means that we selected the simulated state diagram that was (qualitatively) the most similar to the corresponding experimental one. In most cases, we could choose from six simulated diagrams, since there were six pairs of threshold values for which the time course of the angles, hence the state diagrams were computed at each PO value. Representative examples of these comparisons are shown, in graphic form, in Figure 11. The first thing to be noticed is that all experimental state diagrams are of triangular shape before application of the obstacle. This fact restricted the choice of suitable simulated state diagrams to those obtained with *γ*_thr_ = 83°. On the other hand, all three threshold values of *β* are present among the selected simulated state diagrams. The best (qualitative) agreement between experiment and simulation is in the panel pairs A and C. In the former pair of panels, the state diagram is clearly triangular before placement of the obstacle, and *γ* oscillation ceases upon its removal. In panel C, both state diagrams are triangular prior placement of the obstacle but this shape appears to have qualitatively changed after its removal. The two other panel pairs show less conclusive results after removal of the obstacle but the simulated state diagrams still resemble their experimental counterpart. In summary, we could establish good qualitative agreement between experimental and simulated state diagrams.

## Discussion

Search movements constitute an important type of leg movements in insects, in particular, in the stick insect. We set out to model these movements and to try to identify and to explain the underlying mechanisms that produce such movements. To this end, we used a version of our existing one‐leg model of the stick insect (Tóth et al., 2013a,b).

We restricted the leg movement in the model to that in a vertical plane perpendicular to the longitudinal axis of the stick insect, like in the experiments by Berg et al. (2013). Admittedly, this is a considerable constraint on the leg movements, especially when compared to the conditions in the study by Dürr (2001). However, we consider ground contact or the lack of it to be the crucial factor for the absence or presence of search movements (cf. also, Dürr, 2001). The levator‐depressor (coxa‐trochanter) neuromuscular system therefore plays a central role in search movements. But this system remained intact and fully functional both in Berg et al.'s experiments (Berg et al., 2013) and in our model.

We formulated a hypothesis concerning the recovery of the search movements in the stick insect and implemented its details in the model (cf. Results). We found that we could reproduce, qualitatively, many of the search movement patterns that were discerned in the experiments. By so doing, we mimicked the experimental conditions, especially putting an obstacle in the way of the moving leg for a very short period of time and recording the transient time courses of the angles *α*,* β* and *γ*. The qualitative properties we used for comparisons of these time courses were oscillation or the lack of it, trend and kinetics of the oscillatory behavior. Unfortunately, we could not use stronger comparison criteria than these qualitative ones, since the experimental time courses themselves exhibited a wide range of variations in amplitude and frequency of the oscillations during search movements. Thus a statistical description of the results (average values etc.), for example, would not have provided us with useful information on the process of recovery of the search movements in the animal. Moreover, we do, at present, not know anything substantial of the origin of the wild fluctuations seen in the experiments. Their comparison among themselves on a quantitative basis would therefore have proved already impracticable. Because of this, we think that the qualitative comparison between experimental and simulation results we used is justified and sufficient.

The qualitative agreement between experimental and simulation results lets us conclude that the assumptions made in our hypothesis may constitute one possible way of how search movements emerge and are performed in the animal. The central role, here, is played by the levator and depressor neuromuscular network that governs the vertical movement of the femur. Making the rate of recruitment of the fast levator and depressor muscles dependent on the (actual) position of the obstacle, angular time courses in the model, similar to their experimental counterparts, could be produced in the simulations. A very much simplified description of such a mechanism is provided in Figure 7 in form of linear approximations (interpolations). It also enabled us to compute angular time courses at any obstacle position. The mechanisms underlying this dependence are, as yet, unknown but position and tactile sensory signals, most likely, play a part in them. No recruitment mechanism can be present in the extensor‐flexor neuromuscular system, since here there is only a single fast extensor MN (Bässler, 1983; Bässler and Storrer, 1980; Goldammer et al. 2012).

We were led to carry out systematic simulations of search movements over the whole range of obstacle positions (PO values) used in the experiments. This was done with six different pairs of angular threshold values *β*_thr_ and *γ*_thr_, which had been selected such as to enable autonomous periodic leg movements in our existing one‐leg model. The results showed qualitatively the same behavior of the model over large ranges of obstacle positions (Figs. 8 and 9). The oscillations of the *α* and *γ* angles with alternating amplitude, which we encountered during the simulations, might be of particular interest. They may namely hint at the existence of period‐doubling bifurcations in the model, which might eventually lead to chaotic behavior, that is, to disruption of periodic search or stepping movements. At present, it is unclear what the bifurcation parameter might be. We also found isolated positions (PO values) at which the oscillation of all three angles ceased (Fig. 8, bottom right panel). This has not been observed in the experiments, and it is not likely that it reflects a real physiological property. This seems to be some kind of singularity of the model, which, from the practical point of view, can be neglected. A more general point is that the stick insect must be able to perform full search movements at any obstacle position, at least after a transitory period. Some of the simulation results in Figs. 8 and 9 seem to suggest the contrary (e.g., Fig. 8, upper row of panels). However, the appropriate premotor INs can disinhibit the extensor‐flexor MNs in order to revive the oscillatory activity of the EF local network (cf. Fig. 2). Thus the overall movement can show a different pattern. We did not carry out such simulations though in order to keep the effects related to the obstacle positions “clean”, that is, not to confuse them with those of the premotor INs. The results of the systematic simulations do therefore not contradict to the above claim.

State diagrams, produced by plotting the *β* and *γ* angles against each other, seem to contain important information on the type of movement the leg is performing. In several cases, the similarity between experimental and simulation results could only be decided after comparing the corresponding state diagrams (e.g., Figs. 4–6). We found that state diagrams have triangular form during search movements *both* in the experiments and the simulations. Upon removal of the obstacle, this can substantially change, for example, when the *γ* oscillation ceases (Fig. 11A). The qualitative shape of the loops in the state diagram can also change after removal of the obstacle (Fig. 11C), or can remain the same (Fig. 10 right upper panel). In fact, experimental findings show a different shape of the *β*−*γ* diagrams when the animal is stepping on a treadmill (von Uckermann and Büschges, 2009; Berg et al., 2015). They bear some similarity to a quadrilateral one, even though they are not convex, but are markedly different from the triangular‐like ones, seen during search movements.

In the simulations, the triangular shape could only be produced if *γ*_thr_ = 83°. This is the case when the LD system receives a permanent excitation from the EF system (cf. Figs. 2 and 3). This steady excitation keeps the CPG of the LD system in its oscillatory state. The CPG, in turn, drives the EF system periodically. Thus, no load signal or other kind of sensory signal representing ground contact is required for periodic leg movement in this case.

If *γ*_thr_ = 145°, a switch between high and low value of *g*_*γ*_ periodically takes place (cf. Fig. 3). In this case, the *β*−*γ* diagrams have a quadrilateral shape (Fig. 10). Since the state diagram is of triangular shape during search movements, we can assign the threshold value *γ*_thr_ = 83° to this condition. We thus suggest that a sensory signal, generated by the campaniform sensilla upon ground contact, changes this threshold value to *γ*_thr_ = 145° in an as yet unknown way. During stepping, ground contacts periodically occur, hence the threshold value remains the same (*γ*_thr_ = 145°). If, however, during up and down movements of the leg, no ground contact is encountered any more, *γ*_thr_ will change to 83°. The state diagrams (experimental or simulated) thus signify by their shape whether the leg is in “search” or “stepping” mode. The load signal, mostly produced by the campaniform sensilla, plays a crucial part in these processes. Its absence leads to search movements, whereas its presence to proper stepping. This is in good agreement with the findings by Dürr (2001), since he also claims that the absence of ground contact transforms stepping into search movement (Dürr, 2001). The absence of ground contact, in turn, means that the campaniform sensilla are not activated. Hence, their activity or inactivity decides whether the animal performs stepping or search movements.

It is, of course, a rudimentary approximation that we used but a few discrete threshold values to modify the searching behavior in the model. It would seem more natural to assume that the admissible threshold values fill in whole intervals instead. A very recent paper by Szczecinski and Quinn (2017) points exactly in that direction. At present, however, the available data related to this point appears to be insufficient to implement such a version of our model.

Anyway, our results remain, for the time being at least, mainly hypothetical and will need a great deal of further experimental and theoretical support. But even so, they have already proved their physiological relevance by providing a prospective tool to tell apart and characterize different functional modes of an insect leg.

## References

[phy213489-bib-0001] Bässler, U. 1983 Neural basis of elementary behavior in stick insects. Springer Verlag, Berlin‐Heidelberg, Germany.

[phy213489-bib-0002] Bässler, U. 1993 The walking‐ (and searching‐) pattern generator of stick insects, a modular system composed of reflex chains and endogenous oscillators. Biol. Cybern. 69:305–317.

[phy213489-bib-0003] Bässler, U. and J. Storrer . 1980 The neural basis of femur‐tibia‐control‐system in the stick insect *Carausius morosus* I. Motoneurons of the extensor tibiae muscle. Biol. Cybern. 38:607–114.

[phy213489-bib-0004] Berg, E. , A. Büschges , and J. Schmidt 2013 Single perturbations cause sustained changes in searching behavior in stick insects. J. Exp. Biol. 216:1064–1074. 23197090 10.1242/jeb.076406

[phy213489-bib-0005] Berg, E. , SL. Hooper , J. Schmidt , and A. Büschges . 2015 A leg‐local neural mechanism mediates the decision to search in stick insects. Curr. Biol. 25:2012–2017. 26190069 10.1016/j.cub.2015.06.017

[phy213489-bib-0006] Bläsing, B. and H. Cruse 2004a Mechanisms of stick insect locomotion in a gap‐crossing paradigm. J. Comp. Physiol. A 190:173–183. 10.1007/s00359-003-0482-3 14735308

[phy213489-bib-0007] Bläsing, B. and H. Cruse . 2004b Stick insect locomotion in a complex environment: climbing over large gaps. J. Exp. Biol. 207:1273–1286. 15010478 10.1242/jeb.00888

[phy213489-bib-0008] Delcomyn, F. 1987 Motor activity during searching and walking movements of cockroach legs. J. Exp. Biol. 133:111–120. 3430111 10.1242/jeb.133.1.111

[phy213489-bib-0009] Dürr, V. 2001 Stereotypic leg searching movements in the stick insect: kinematic analysis, behavioural context and simulation. J. Exp. Biol. 204:1589–1604. 11398748 10.1242/jeb.204.9.1589

[phy213489-bib-0010] Goldammer J. A. Büschges , and J. Schmidt . 2012 Motoneurons, dum cells, and sensory neurons in an insect thoracic ganglion: a tracing study in the stick insect *Carausius morosus* . J. Comp. Neurol. 520:230–257. 21618233 10.1002/cne.22676

[phy213489-bib-0011] Hooper S. L. , C. Guschlbauer , M. Blümel , P. Rosenbaum , M. Gruhn , T. Akay , and A. Büschges . 2007 Neural control of unloaded leg posture and leg swing in stick insect, cockroach, and mouse differs from that in large animals. J. Exp. Biol. 210:1092–1108. 19339606 10.1523/JNEUROSCI.5510-08.2009 PMC6665391

[phy213489-bib-0012] Karg, G. , G. Breuel , and U. Bässler 1991 Sensory influences on the coordination of two leg joints during searching movements of stick insects. Biol. Cybern. 64:329–335.

[phy213489-bib-0013] Pearson, K. G. 1972 Central programming and reflex control of walking in the cockroach. J. Exp. Biol. 56:173–193.

[phy213489-bib-0014] Pick, S. and R. Strauss . 2005 Goal‐driven behavioral adaptations in gap‐climbing drosophila. Curr. Biol. 15:1473–1478. 16111941 10.1016/j.cub.2005.07.022

[phy213489-bib-0015] Schütz, C. and V. Dürr . 2011 Active tactile exploration for adaptive locomotion in the stick insect. Philos. Trans. Royal Soc. London B 366:2996–3005. 10.1098/rstb.2011.0126 PMC3172591 21969681

[phy213489-bib-0016] Szczecinski, N. and R. D. Quinn . 2017 Leg‐local neural mechanisms for searching and learning enhance robotic locomotion. Biol. Cybern. 1–14. DOI 10.1007/s00422‐017‐0726‐x. [Epub ahead of print] 28782078 10.1007/s00422-017-0726-x

[phy213489-bib-0017] Tóth, T. I. , J. Schmidt , A. Büschges , and S. Daun‐Gruhn . 2013a A neuro‐mechanical model of a single leg joint highlighting the basic physiological role of fast and slow muscle fibres of an insect muscle system. PLOS ONE 8:e78247. 24244298 10.1371/journal.pone.0078247 PMC3823925

[phy213489-bib-0018] Tóth, T. I. , M. Grabowska , J. Schmidt , A. Büschges , and S. Daun‐Gruhn . 2013b A neuro‐mechanical model explaining the physiological role of fast and slow muscle fibres at stop and start of stepping of an insect leg. PLOS ONE 8:e78246. 24278108 10.1371/journal.pone.0078246 PMC3838373

[phy213489-bib-0019] von Uckermann, G. and A. Büschges . 2009 Premotor interneurons in the local control of stepping motor output for the stick insect single middle leg. J. Neurophysiol. 102:1956–1975. 19605613 10.1152/jn.00312.2009

[phy213489-bib-0020] Zill, S. N. , J. Schmitz , and A. Büschges . 2004 Load sensing and control of posture and locomotion. Arthropod Struct. Dev. 33:273–286. 18089039 10.1016/j.asd.2004.05.005

[phy213489-bib-0021] Zill, S. N. , A. Büschges , and J. Schmitz . 2011 Encoding of force increases and decreases by tibial campaniform sensilla in the stick insect, *Carausius morosus* . J. Comp. Physiol. 197:851–867. 21544617 10.1007/s00359-011-0647-4

